# Iatrogenic Creutzfeldt-Jakob Disease from Commercial Cadaveric Human Growth Hormone

**DOI:** 10.3201/eid1904.121504

**Published:** 2013-04

**Authors:** Brian S. Appleby, Mei Lu, Alberto Bizzi, Michael D. Phillips, Sally M. Berri, Madeleine D. Harbison, Lawrence B. Schonberger

**Affiliations:** Cleveland Clinic Foundation, Cleveland, Ohio, USA (B.S. Appleby, M. Lu, M.D. Phillips);; Instituto Clinico Humanitas,Milan, Italy (A. Bizzi);; Case Western Reserve University, Cleveland (S.M. Berri);; Mount Sinai School of Medicine, New York, New York, USA (M.D. Harbison);; Centers for Disease Control and Prevention, Atlanta, Georgia, USA (L.B. Schonberger)

**Keywords:** Creutzfeldt-Jakob disease, iatrogenic Creutzfeldt-Jakob disease, human growth hormone, case report, commercial, cadaveric, prions and related diseases

**To the Editor:** Iatrogenic Creutzfeldt-Jakob disease (iCJD) is an acquired form of prion disease that has been declining in incidence since the mid-1990s (*1*). Worldwide, at least 226 cases of iCJD, including 29 US cases, have been associated with administration of contaminated human growth hormone (hGH) from cadavers. Reported incubation periods ranged from 5 to 42 years (mean 17 years) (*2*). Commercially produced cadaveric hGH has been associated with only 1 previously reported case of iCJD: CJD developed in a 39-year-old Austrian man ≈22 years after he received commercial cadaveric hGH (Crescormon, Kabivitrum, Stockholm, Sweden) during 1984–1985 (*3*). We report a second case of probable iCJD acquired through treatment with commercial cadaveric hGH.

The patient was born at 32 weeks’ gestation with subsequent developmental delay, agenesis of the corpus callosum, and panhypopituitarism. He demonstrated clinical and laboratory signs of growth hormone deficiency but was denied treatment with hGH through the US government–supported National Hormone and Pituitary Program (NHPP) because he did not meet the height requirement. Treatment with commercial cadaveric hGH began when he was 5.8 years of age and continued for 23 months (1983–1985). He received 1.5 units intramuscularly 3× per week and was primarily treated with Asellacrin (Ares-Serono, Geneva, Switzerland). In early 1984, for an unspecified duration, he received Crescormon (Kabivitrum) because of an Asellacrin shortage. Treatment was halted in 1985 because of iCJD concerns and resumed 2 years later with recombinant hGH.

At age 33, 26.5 years (range 25.5–28 years) after the midpoint of commercial cadaveric hGH treatment, dizziness and gait imbalance developed, causing a fall. The patient’s mental status also began declining, and he never returned to his baseline status. Six months after illness onset, he experienced hallucinations, weakness of lower extremities, and limb ataxia. Seven months after the fall, he entered a state of akinetic mutism; he died 9 months after symptom onset. A lumbar puncture, performed 8 months after illness onset, demonstrated 14-3-3 proteins and an elevated cerebrospinal fluid (CSF) τ level of 14,111 pg/mL (decision point 1,150 pg/mL) (*4*), although the specimen was contaminated with blood (39,375 erythrocytes/μL). Electroencephalogram demonstrated severe diffuse encephalopathy. Two brain magnetic resonance imaging studies performed 8 months after illness onset indicated probable CJD, given lack of prior metabolic and anoxic insults (Figure). The patient was discharged from a referral hospital with this diagnosis; no postmortem analysis was conducted.

**Figure:**
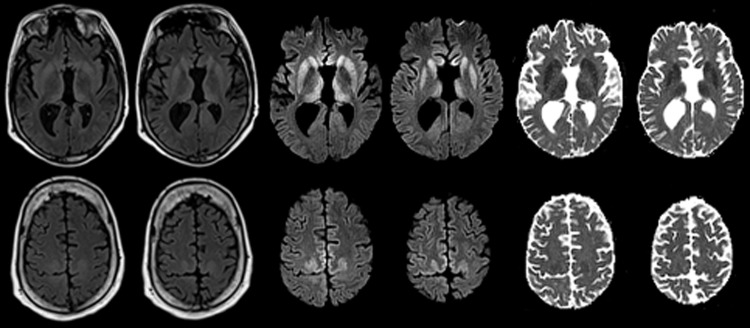
Maps showing axial fluid attenuated inversion recovery (FLAIR), diffusion-weighted imaging (DWI), and apparent diffusion coefficient (ADC) at the level of the basal nuclei (top row) and dorsal frontoparietal cortex (bottom row) of the brain of a 33.8-year-old man with agenesis of the corpus callosum, schizencephaly, and heterotopia. Note the symmetrical DWI signal hyperintensities in the striatum and dorsomedial part of the thalami. In addition, DWI signal hyperintensities occurred in the cingulate, precuneus and in the dysplastic gray matter along the anterior lips of the schizencephalic clefts at the level of the precentral gyri. The signal abnormalities are associated with decreased diffusivity on ADC maps and are much less prominent on FLAIR images. These findings are highly suggestive of Creutzfeldt-Jakob disease.

On the basis of World Health Organization criteria, we conclude that this patient had probable iCJD as a result of hGH treatment (*5*). The patient’s condition was treated with 2 different formulations of commercial cadaveric hGH, including one of the same brands in the same year as that of the first reported patient with iCJD associated with commercial cadaveric hGH (*3*). The patient’s incubation period (25.5–28 years) is well within expectations (*1*).

Despite an ongoing active surveillance program that identified ≈3,500 of ≈4,500 post-1977 cadaveric hGH recipients in the US NHPP, all 29 CJD infections in NHPP recipients occurred among the estimated ≈2,700 pre-1977 recipients (*1*,*2*). This significant reduction in iCJD was attributed to the 1977 introduction of a highly selective, column chromatography step in the hormone purification protocol that can markedly reduce prion infectivity (*1*,*2*). As shown by the many iCJD cases linked to hGH in France, the efficacy of column chromatography purification steps may vary (*1*). Commercially derived cadaveric hGH was produced in different laboratories from those that produced NHPP-distributed hGH, and sufficient details regarding sourcing and production methods of the commercial products are lacking. Approximately 10,000 persons, mostly outside the United States, received commercial cadaveric hGH produced by Kabivitrum, and substantially fewer persons received product from Ares-Serono (A.F. Parlow, pers. comm.). Identification through passive surveillance of 2 CJD cases among recipients of such hGH further supports a causal, rather than chance, association between commercial hormone and CJD. It also suggests a difference in iCJD risk between post-1977 NHPP-distributed hGH and commercial cadaveric hGH.

Limitations of this report include the lack of neuropathologic confirmation and insufficient information to strongly implicate a single commercial cadaveric hGH product as infection source. The report of another iCJD case-patient who received Crescormon during the same period provides some evidence that the product was the source of prion contamination. Although the patient may have had sporadic CJD, his young age at disease onset (33 years) makes this unlikely (*6*).

This report suggests that a potential risk for iCJD in persons who received commercial cadaveric hGH should be considered. Also, clinicians should not assume that all cadaveric hGH administered after 1977 carries the same risk for infectivity. In addition, when CJD is being considered as a clinical diagnosis, a history of exposure to cadaveric hGH should always be sought, even when patients have normal or tall stature. Finally, we recommend that when a clinical diagnosis of CJD is suspected, but before the patient’s death, the local caregivers, with the family, should initiate arrangements for a postmortem examination to confirm diagnosis (e.g., www.cjdsurveillance.com).
